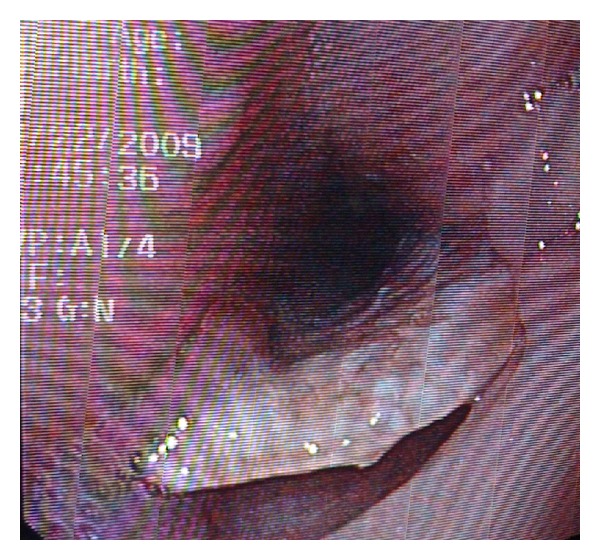# An Unusual Case of Tracheoesophageal Fistulae

**DOI:** 10.1155/2012/524687

**Published:** 2012-06-28

**Authors:** Jadelis Giquel, Christina Matadial, Yiliam F. Rodriguez Blanco, Ricardo Martinez-Ruiz, Dao Nguyen, Keith Candiotti

**Affiliations:** ^1^Department of Anesthesiology, University of Miami Miller School of Medicine and Veterans Hospital 1611 NW 12 Ave Miami, FL 33125, USA; ^2^Department of Surgery, University of Miami Miller School of Medicine, 1611 NW 12 Ave Miami, FL 33125, USA; ^3^Department of Anesthesiology and Internal Medicine, University of Miami Miller School of Medicine, 1611 NW 12 Ave Miami, FL 33125, USA

## Abstract

Acquired tracheoesophageal fistulae (TEF) are commonly due to malignancy (M. F. Reed and D. J. Mathisen, 2003). We present the case of a patient with a deceptive history for TEF and report an approach that provides adequate oxygenation, ventilation, surgical exposure, and postoperative analgesia with excellent outcome.

## 1. Introduction

Acquired tracheoesophageal fistulae (TEF) are most commonly due to malignancy [1]. Benign fistulae are uncommon [1, 2]. There are fewer than 10 reported cases in the English literature describing benign tracheoesophageal fistula formation secondary to a swallowed dental prosthesis [3]. Presentation may range from subclinical to severe respiratory distress [3]. The index of suspicion is increased if the patient also presents with acute dysphagia. Diagnosis may be delayed if the patient presents only with respiratory symptoms [4–6].

We present the case of a 45-year-old male with a history of recurrent pneumonias over a period of months. He ultimately underwent a thoracotomy for a TEF secondary to foreign body lodged first in his esophagus that eroded over time into his airway.

## 2. Case Report

**Figure 1:**
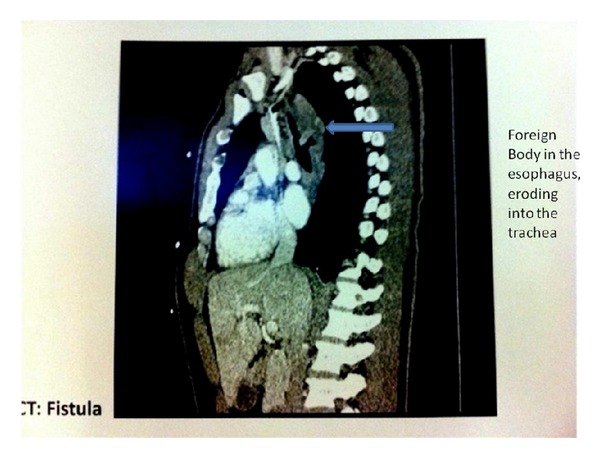


A 45-year-old male was transferred to our facility from an outside institution with the diagnosis of TEF and the presence of a possible foreign body seen on CT scan of his chest. The patient gave a history of recurrent pneumonia and cough in the last few months. Prior to presentation to the hospital, the patient had finished a course of antibiotics. Despite this, he complained of increasing cough over the prior week but no history of dysphagia, hemoptysis, hematemesis, or chest pain. The physical exam was unremarkable except for inspiratory crackles and diminished breath sounds heard at the lung bases. Axial CT images of the chest both with and without intravenous contrast were obtained. A small, linear, walled air collection was noted just above the carina tracking towards the esophagus, a finding suspicious for a tracheoesophageal fistula (Figure 1).

Under sedation and spontaneous ventilation, bronchoscopic and endoscopic examinations were performed. A hard, disc-shaped foreign body was impacted in the esophagus and was also protruding into the lumen of the distal trachea (see Figure 2). Gentle attempts to remove the FB were unsuccessful and aborted. Two days later the patient underwent a thoracotomy for foreign body removal and TEF repair. Mask ventilation was easy with low positive inspiratory pressures. A left double lumen tube was placed via direct laryngoscopy. With fiberoptic bronchoscope (FOB) allowing for direct vision, the bronchial lumen was advanced into the left main stem bronchus with subsequent deflation of the right lung. With the double lumen ETT in position, ventilation of only the left lung avoided any air insufflations through the fistula into the esophagus (due to the anatomical location of the fistula 1 cm above the carina). The patient was placed in the left lateral decubitus position and the ETT placement again confirmed with the FOB. Surgical approach was via right thoracotomy, with removal of the foreign body and primary repair of both the trachea and the esophagus. After the surgery the patient resumed spontaneous ventilation and was extubated prior to transfer to the ICU. Pathology confirmed that the foreign body was a dental plate. Later, the patient revealed that after an appendectomy 5 years ago, he could not find his partial dental plate.

## 3. Discussion

Diagnosing impacted dentures or any foreign body can be difficult when patients present late, often with very few reliable clinical signs. In our case, the patient did not exhibit any signs or symptoms of a foreign body other than frequent episodes of pneumonia.

Acquired TEF is rare and is mainly described in the adult population as a result of trauma, corrosive ingestion, foreign body, inflammatory process, or malignancy [7]. The anesthetic management for repair of TEF, whether congenital or acquired, is a significant challenge for the anesthesiologist [8]. Common problems include difficulty with oxygenation and/or ventilation resulting from placement of the ETT in or above the fistula with subsequent gastric dilatation, atelectasis, or pulmonary changes related to recurrent aspiration. Standard anesthetic management includes awake tracheal intubation with avoidance of muscle relaxants and positive pressure ventilation until the fistula is controlled to prevent isufflation of the esophagus or the stomach as well as to facilitate surgical identification of the fistula [8, 9]. The surgeon and anesthesiologist are competing for airway access. The site and size of the lesion must be carefully noted as this may dictate the anesthesiologist's approach. Fortunately, most TEFs presenting for surgical repair are in the upper two-thirds of the trachea. If the tip of the ETT lies above the TEF, gastric dilatation and aspiration can occur. Intubating the lumen of a large TEF with failure to ventilate is a major concern. The choice must be made to isolate only one lung or to use an alternative. Carinal and bronchial TOF are rare but can present the anesthetist with major difficulties. The site of the lesion means that protection of both lungs with a standard tracheal tube will be impossible. Once the TEF is isolated, ventilation can continue without fear of soiling and gastric dilatation. There are reports in the literature of TEFs being repaired with the patient breathing spontaneously, although assisted ventilation was often necessary. Immediate extubation is the goal, avoiding unnecessarily stressing the surgical repair [9].

It has been shown that postoperative positive pressure ventilation is associated with an increased incidence of anastomotic breakdown and stenosis, especially after tracheal resection [10–13].

Repair of an acquired TEF may present multiple difficulties to both the anesthesiologist and surgeon [8, 9]. We report an approach that provides adequate oxygenation, ventilation, surgical exposure and postoperative analgesia with excellent outcome.

In conclusion, the unnoticed swallowing of items of odontogenic origin, though infrequent, can potentially be dangerous. If it is suspected that an anesthetized patient has swallowed a foreign body, the appropriate medical specialist should be consulted, as it may be necessary to identify and remove an object with sharp edges to avoid late complications that may require surgical intervention. Attention must be paid to patients at increased risk of unnoticed foreign body ingestion. This includes young children and patients with comorbidities that limit cognition.

## Figures and Tables

**Figure 2:**